# Assessment and associated factors of comprehensive HIV knowledge in an at-risk population: a cross-sectional study from 19,286 young persons in Nigeria

**DOI:** 10.1177/20499361231163664

**Published:** 2023-04-04

**Authors:** Yusuf Olushola Kareem, Cyprian Issahaku Dorgbetor, Edward Kwabena Ameyaw, Zubaida Abubakar, Babatunde Adelekan, Erika Goldson, Ulla Mueller, Oyelola Adegboye

**Affiliations:** United Nations Population Fund, Abuja, Nigeria; Ghana Health Service, Municipal Health Directorate, Techiman, Ghana; Institute of Policy Studies and School of Graduate Studies, Lingnan University, Tuen Mun, Hong Kong; United Nations Population Fund, Abuja, Nigeria; United Nations Population Fund, Abuja, Nigeria; United Nations Population Fund, Abuja, Nigeria; United Nations Population Fund, Abuja, Nigeria; Public Health and Tropical Medicine, Medical and Veterinary Sciences, James Cook University, 1 James Cook Drive, Douglas, Queensland 4814, Australia; Australian Institute of Tropical Health and Medicine, James Cook University, 1 James Cook Drive, Douglas, Queensland 4814, Australia

**Keywords:** adolescents, comprehensive HIV knowledge, contraceptive, HIV, Nigeria, young persons

## Abstract

**Background:**

The prevalence of HIV among young people aged 15–19 years in Nigeria is estimated as 3.5%, the highest among West and Central African countries. Comprehensive knowledge of HIV is associated with increased awareness of preventive interventions and a reduction in the spread of HIV. Therefore, this article seeks to assess and determine the associated factors of comprehensive HIV knowledge among youths in Nigeria.

**Methods:**

The study used the 2018 Nigerian Demographic Health Survey, a cross-sectional survey that employed a two-stage cluster sampling method. Comprehensive knowledge of HIV was assessed based on five questions. The data were analysed separately for men and women aged 15–24 years. A multivariable log-binomial regression model was used to determine factors associated with comprehensive HIV knowledge. All analysis was performed using Stata 15.0 and adjusted for weighting, clustering and stratification.

**Results:**

A total of 15,267 women and 4019 men aged 15–24 years were included in this study. The prevalence of comprehensive knowledge of HIV was higher among women than among men (42.6% *versus* 33.7%; *p* < 0.001) and lower among younger ages 15–17 years compared with other ages. The findings revealed that age, ethnicity, wealth, education and exposure to mass media were statistically significant factors associated with comprehensive knowledge of HIV. In addition, religion, place of residence, phone ownership, internet use, currently working and having initiated sex were significant factors among women and modern contraceptive use among men.

**Conclusion:**

Key findings from this study imply that public health programmes in Nigeria should focus on providing information on HIV/AIDS using different approaches, including comprehensive sex education as well as health promotion and education strategies in the formal and informal sectors. Because media exposure is a common and cost-effective way of public health promotion and education in modern times, emphasis could also be placed on using this channel to reach the target population.

## Background

HIV continues to be a major global health issue.^1^ Since the emergence of the HIV epidemic, about 75 million people have been infected, with about 32 million deaths.^1,2^ Approximately 37.7 million people were living with HIV at the end of 2020, with 1.5 million people becoming newly infected with HIV globally, with African countries accounting for about 60% of all cases.^1^ In 2012, about 2.1 million (5.9%) people living with HIV were young men and women.^3^ Several risky sexual behaviours such as early sexual initiation (before 18 years), having multiple sexual partners and unprotected sexual intercourse, having a high-risk partner and engaging in sex work were critical factors putting young people at higher risk of HIV infection.^3^ In 2019, it was recorded that two out of every seven new HIV infections occurred among young people globally.^4^

In sub-Saharan Africa (SSA), while much strides have been made in HIV prevention and treatment, incidence rates are increasing among the youthful population. For example, more than 80% of the young people infected with HIV reside in SSA.^4,5^ Worldwide, 42%, of which only one in five adolescent girls in SSA is aware of her HIV status.^3,4^ These statistics suggest the extent to which young people in SSA, including Nigeria, are susceptible to HIV/AIDS and its ramifications.

In Nigeria, the prevalence of HIV among young people is estimated to be 3.5%, the highest among countries in West and Central Africa.^6^ The 2018 Nigeria Demographic and Health Survey (NDHS) reported that 89.5% of girls and 89.3% of boys aged 15–19 had heard of AIDS. On HIV prevention measures, 51.6% of girls, relative to 63% of boys, knew that consistency in condom use could reduce the risk of HIV infection.^6^ The 2020 condom accessibility and use in Nigeria poll conducted by the National Agency for the Control of AIDS and AIDS Healthcare Foundation reported that about one in three Nigerians used a condom during sex, and only one in four uses condoms for protection against sexually transmitted diseases (STDs).^7^ Evidence from the Nigeria HIV/AIDS Indicator and Impact Survey (NAIIS) shows Nigeria could not achieve the 90-90-90 targets.^8^ The Integrated Biological and Behavioural Surveillance (IBBS) Survey report revealed some key populations with a high prevalence of HIV.^9^ These included men who have sex with men (MSM) (22.9%), women who sell sex (19.4%) and people who inject drugs (3.4%).^9^

Despite the efforts by stakeholders, inadequate HIV knowledge among young men and women coupled with sociocultural factors may contribute to stigmatising tendencies towards those infected and affected by HIV. If not addressed, stigmatisation and discrimination may continue, especially against young men and women, which could hinder them from testing and adhering to treatment.^10,11^ Comprehensive knowledge of HIV and sexually transmitted infection (STI) transmission pathways are associated with increased awareness of preventive interventions and reduced spread of HIV/STI.^3,11–13^ Although knowledge is only one component of health literacy, previous research has highlighted the positive association between HIV-related knowledge and overall health literacy levels.^12^

It is, therefore, imperative to consider improving comprehensive HIV knowledge, an essential strategy for ending HIV and AIDS by 2030, particularly among this at-risk population which forms a higher proportion of the populace. HIV/AIDS is currently the leading cause of death among adolescents in Africa, and the second leading cause of adolescent death worldwide, with SSA having the highest fatalities.^10^ Therefore, it is prudent to investigate what young people know about HIV to address misconceptions associated with the virus. Understanding and addressing HIV knowledge gaps among youth is crucial when designing behaviour change interventions.^10^

This study aims to assess and determine the associated factors of comprehensive HIV knowledge among young people in Nigeria.

## Methods

### Data source and sampling strategy

This study was based on a publicly available dataset from the NDHS conducted in 2018. Demographic and Health Surveys (DHS) have been conducted in more than 90 countries since 1984 using validated tools.^14,15^ A total of 40,427 households were interviewed, consisting of 41,821 women aged 15–49 years from the women’s questionnaire and 13,311 men aged 15–59 years from the men’s questionnaire. Both women’s and men’s questionnaires yielded a response rate of 99%. For this study, we extracted information on male and female adolescents aged 15–19 years and young adults aged 20–24 years (youths). The sampling method for the 2018 NDHS was a two-stage stratified sampling; the 36 states and the Federal Capital Territory were stratified into urban and rural areas. Then, the first sampling stage was the selection of 1400 Enumeration Areas (EAs), and the next stage was interviewing residents or visitors who stayed in the selected households the night before the survey.^14^

### Dependent variable

The dependent variable was comprehensive knowledge of HIV, which was measured using three questions on the knowledge about HIV and two questions about local misconceptions, which are incorrect beliefs about HIV transmission. These variables are (1) the knowledge about the consistent use of condoms during sexual intercourse, (2) knowing that having one uninfected faithful partner could reduce the chance of contracting HIV and (3) knowing that a healthy-looking person can have HIV; (4) knowledge about the misconception that HIV can be transmitted through mosquitoes, as well as (5) misconception that HIV can be transmitted by sharing of food with someone infected with HIV. Respondents who answered all these questions correctly were assigned 1, implying having comprehensive knowledge of HIV, and 0, if any of these questions were answered incorrectly.

### Independent variables

The following respondents’ background characteristics were used as independent variables: (1) current use of modern contraceptive methods (no *versus* yes) – including male and female sterilisation, injectables, intrauterine devices (IUDs), contraceptive pills, implants, female and male condoms, the standard days method, the lactational amenorrhea method (LAM) and emergency contraception and any other modern method including diaphragm, contraceptive jelly or foam, (2) age group (15–17, 18–19 and 20–24 years), (3) region (North Central, North East, North West, South East, South South and South West), (4) place of residence (urban *versus* rural), (5) wealth quintiles – composite index to measure socioeconomic status of households using information on assets, goods and services, dwelling and housing conditions and operationalised using the principal component analysis was divided into quintiles (poorest, poorer, middle, richer and richest), (6) education (no formal education, primary, secondary and above), (7) owning a mobile phone (yes *versus* no), (8) exposure to mass media (means of communication to reach a large audience) – TV, radio, newspaper or magazine (yes *versus* no), (9) having access to the internet (yes *versus* no), (10) covered by health insurance (yes *versus* no), (11) religion (Catholics, other Christians, Islam and other religion), (12) ethnicity (Fulani, Hausa, Igbo, Yoruba and other ethnic minorities), (13) marital status (never *versus* ever married), (14) sex of household head (male *versus* female), (15) currently working (yes *versus* no) and (16) ever had sexual intercourse (yes *versus* no).

### Data analysis

The descriptive statistics of variables were presented using frequencies and percentages separated by sex. The prevalence of HIV comprehensive knowledge with Clopper–Pearson’s 95% confidence intervals (CIs) was computed by respondents’ background characteristics and across states. Differences in the prevalence of HIV comprehensive knowledge were assessed using a two-sided *z*-test of difference in proportion. Univariate and multivariable log-binomial regression models were used to determine the risk factors associated with comprehensive HIV knowledge separately for men and women. The results were presented as crude risk ratio (cRR) for unadjusted models and adjusted risk ratio (aRR) together with their 95% CIs. A multicollinearity test does not reveal any collinearity using the variance inflation factor. All analyses were performed using Stata 15.0 (StataCorp LP, College Station, Texas, USA), adjusting for the complex survey design – weighting, clustering and stratification and at a 5% significance level

## Results

Of the 55,132 respondents who completed the survey interview for both men and women, 19,286 were youth aged 15–24 years. Table 1 presents the description of the study participants by their background characteristics. Although there were more women, 15,267 (79.2%), than men, 4019 (20.8%), their sociodemographic characteristics were generally similar (e.g. wealth index, mass media exposure, ethnicity, religion, health insurance coverage). Men were younger (40.7% *versus* 34.3% aged 15–17 years), and about 3 in 10 respondents were from North West, Nigeria. Most respondents were Muslims (about three in five) and resided in rural areas, while 7 in 10 had at least a secondary education. About 46% of women owned a mobile phone compared with 59.5% of men. Fewer women had access to the internet (18.9% *versus* 37.3%) and were currently working (44.5% *versus* 64%) compared with men. About three in five female respondents had initiated sex compared with one in four men. Only 6.3% of the male respondents were married compared with 42.7% of women. Women-headed households were less than one in five. Noticeably, a higher proportion of men (11.3%) use modern contraceptive methods compared with 5.3% of women. However, 76.1% of men, compared with 46.1% of women of the sample, have initiated sexual intercourse.

**Table 1.** Characteristics of study participants by sex.

Variables	Female, *n* (%)	Male, *n* (%)
Had comprehensive HIV knowledge		
No	8763 (57.4)	2665 (66.3)
Yes	6504 (42.6)	1354 (33.7)
Current use of modern contraceptive		
No	14,448 (94.6)	3563 (88.7)
Yes	819 (5.4)	456 (11.3)
Age group		
15–17	5236 (34.3)	1637 (40.7)
18–19	3203 (21.0)	860 (21.4)
20–24	6828 (44.7)	1522 (37.9)
Region		
North Central	2223 (14.6)	585 (14.6)
North East	2693 (17.6)	768 (19.1)
North West	4953 (32.4)	1304 (32.4)
South East	1584 (10.4)	391 (9.7)
South South	1575 (10.3)	449 (11.2)
South West	2239 (14.7)	523 (13.0)
Place of residence		
Urban	6730 (44.1)	1717 (42.7)
Rural	8537 (55.9)	2302 (57.3)
Wealth quintiles		
Poorest	2625 (17.2)	829 (20.6)
Poorer	3171 (20.8)	810 (20.2)
Middle	3135 (20.5)	829 (20.6)
Richer	3326 (21.8)	860 (21.4)
Richest	3010 (19.7)	691 (17.2)
Education		
None	4378 (28.7)	830 (20.6)
Primary	1599 (10.5)	383 (9.5)
Secondary and above	9290 (60.9)	2806 (69.8)
Own a phone		
No	8243 (54.0)	1627 (40.5)
Yes	7024 (46.0)	2392 (59.5)
Exposure to mass media		
No	5309 (34.8)	1424 (35.4)
Yes	9958 (65.2)	2595 (64.6)
Access to internet		
No	12,381 (81.1)	2519 (62.7)
Yes	2886 (18.9)	1500 (37.3)
Covered by health insurance		
No	15,005 (98.3)	3988 (99.2)
Yes	262 (1.7)	31 (0.8)
Religion		
Catholics	1508 (9.9)	394 (9.8)
Other Christians	4851 (31.8)	1191 (29.6)
Islam	8837 (57.9)	2409 (59.9)
Others	71 (0.5)	25 (0.6)
Ethnicity		
Fulani	1021 (6.7)	254 (6.3)
Hausa	5079 (33.3)	1493 (37.2)
Igbo	1986 (13.0)	485 (12.1)
Yoruba	2061 (13.5)	483 (12.0)
Others	5120 (33.5)	1303 (32.4)
Marital status		
Never married	8753 (57.3)	3765 (93.7)
Ever married	6514 (42.7)	254 (6.3)
Sex of household head		
Male	12,614 (82.6)	3466 (86.2)
Female	2653 (17.4)	553 (13.8)
Currently working		
No	8478 (55.5)	1448 (36.0)
Yes	6788 (44.5)	2571 (64.0)
Ever had sex		
No	6424 (42.1)	3061 (76.1)
Yes	8842 (57.8)	958 (23.9)
Overall	15,267 (100)	4019 (100)

**Figure 1.**
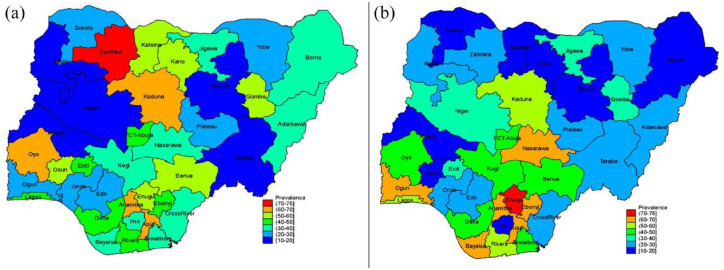
Spatial map of comprehensive HIV education among Nigerian youths. (a) Female. (b) Male.

The comprehensive knowledge about HIV varies by state (Figure 1(a) and (b)). About 7–10 female youth in Zamfara state had comprehensive HIV knowledge, compared with about 2 in 10 women in Kebbi, Niger, Bauchi and Taraba states. Similarly, nearly 2 in 10 men had comprehensive HIV knowledge in Bauchi, Kano, Kwara, Imo, Sokoto, Katsina and Borno states, whereas the highest was reported in Enugu state, where almost 7 in 10 young men had comprehensive HIV knowledge.

The prevalence of comprehensive knowledge of HIV and their 95% CI are presented in Table 2. Overall, the prevalence of comprehensive knowledge of HIV differs by sex [prevalence among women (*P_F_*) = 42.6% *versus* prevalence among men (*P_M_*) = 33.7%; *P_F_* − *P_M_* = 8.9, *p* < 0.001] and sociodemographic indicators. For example, comprehensive knowledge of HIV was significantly higher among women than among men aged 15–17 years (*P_F_* = 35.5% *versus P_M_* = 25.5%; *P_F_* − *P_M_* = 10.0, *p* < 0.001), 18–19 years (*P_F_* = 42.5% *versus P_M_* = 34.9%; *P_F_* − *P_M_* = 7.6, *p* < 0.001) and those aged 20–24 years (*P_F_* = 48.1% *versus P_M_* = 41.9%; *P_F_* − *P_M_* = 6.2, *p* < 0.001). There was no significant sex difference in comprehensive HIV knowledge among youths in a female-headed household (*P_F_* = 42.6% *versus P_M_* = 33.7%; *P_F_* − *P_M_* = 4.2, *p* = 0.069); however, a significant sex difference was observed for youths in male-headed households (*P_F_* = 32.9% *versus P_M_* = 38.7%; *p* = 0.024). Young females who had access to mass media, compared with those who did not, had a higher prevalence of comprehensive HIV knowledge than men [*P_F_* = 48.2 (95% CI: 46.5–49.8) *versus P_M_* = 40.6 (95% CI: 38.1–43.1)]. Also, comprehensive HIV knowledge was significantly higher for young females (*P_F_* = 40.7, 95% CI: 38.9–42.5) with no sexual experience compared with men (*P_M_* = 9.7, 95% CI: 27.6–31.8). The highest significant sex difference in comprehensive HIV knowledge in Nigeria’s six geographical regions was observed in North West (*P_F_* − *P_M_* = 24.6, *p* < 0.0001). Other regional significant sex differences were observed in North Central (*P_F_* − *P_M_* = −7.7, *p* < 0.0001), North East (*P_F_* − *P_M_* = 7.7, *p* < 0.001) and South West (*P_F_* − *P_M_* = 5.6, *p* < 0.001). Similarly, the prevalence of comprehensive HIV knowledge differs significantly between female and male Hausa (*P_F_* − *P_M_* = 21.5, *p* < 0.001), Fulani (*P_F_* − *P_M_* = 9.3, *p* < 0.05) and Yoruba (*P_F_* − *P_M_* = 5.8, *p* < 0.05) ethnic groups. Although comprehensive HIV knowledge was lower among women compared with men, no statistically significant difference was observed (55.3% *versus* 58.3%, *p* = 0.301).

**Table 2.** Prevalence of HIV comprehensive knowledge among young women and men by background characteristics.

Variable	Comprehensive HIV knowledge		
	Female	Male	Difference (%)
	*P_F_* (95% CI)	*P_M_* (95% CI)	*P_F_* − *P_M_* (*p* value)^a^
Overall	42.6 (41.3–44.0)	33.7(31.8–35.8)	8.9 (<0.001)
Current use of modern contraceptive			
No	41.9(40.6–43.2)	30.6(28.6–32.6)	11.3 (<0.001)
Yes	55.3(51.0–59.5)	58.3(52.5–63.9)	3.0 (0.301)
Age group			
15–17	35.5 (33.8–37.3)	25.5 (22.8–28.4)	10.0 (<0.001)
18–19	42.5 (40.3–44.7)	34.9 (31.4–38.6)	7.6 (<0.001)
20–24	48.1 (46.3–50.0)	41.9 (39.0–44.9)	6.2 (<0.001)
Region			
North Central	32.4 (29.6–35.4)	39.4 (34.5–44.5)	−7.0 (<0.05)
North East	28.6 (25.8–31.5)	20.9 (17.6–24.6)	7.7 (<0.001)
North West	51.3 (48.8–53.7)	26.7 (23.5–30.2)	24.6 (<0.001)
South East	53.0 (49.8–56.1)	53.7 (47.8–59.5)	−0.7 (0.804)
South South	39.5 (36.0–43.0)	44.3 (38.6–50.2)	4.8 (0.068)
South West	45.4 (41.4–49.5)	39.8 (33.2–46.9)	5.6 (<0.05)
Place of residence			
Urban	51.4 (49.1–53.6)	41.6 (38.3–45.1)	9.8 (<0.001)
Rural	35.7 (34.0–37.5)	27.9 (25.6–30.3)	7.8 (<0.001)
Wealth quintiles			
Poorest	30.1 (27.2–33.2)	15.2 (12.1–19.0)	14.9 (<0.001)
Poorer	35.0 (32.7–37.5)	27.1 (23.7–30.9)	7.9 (<0.001)
Middle	41.9 (39.2–44.8)	33.6 (29.8–37.6)	8.3 (<0.001)
Richer	48.0 (44.7–51.2)	40.8 (36.0–45.7)	7.2 (<0.001)
Richest	56.3 (53.7–58.9)	55.1 (49.7–60.4)	1.2 (0.567)
Education			
None	31.0 (28.6–33.4)	16.2 (12.8–20.4)	14.8 (<0.001)
Primary	33.8 (30.7–37.0)	21.2 (16.9–26.2)	12.6 (<0.001)
Secondary and above	49.6 (47.9–51.4)	40.6 (38.2–43.1)	9.0 (<0.001)
Own a mobile phone			
No	34.6 (33.2–36.2)	24.7 (22.2–27.4)	9.9 (<0.001)
Yes	52.0 (50.1–53.8)	39.9 (37.3–42.5)	12.1 (<0.001)
Exposure to mass media			
No	32.2 (30.3–34.1)	21.3 (18.5–24.3)	10.9 (<0.001)
Yes	48.2 (46.5–49.8)	40.6 (38.1–43.1)	7.6 (<0.001)
Access to internet			
No	38.9 (37.5–40.3)	26.3 (24.2–28.5)	12.6 (<0.001)
Yes	58.5 (55.4–61.6)	46.2 (42.8–49.7)	12.3 (<0.001)
Covered by health insurance			
No	42.2 (40.9–43.6)	33.5 (31.6–35.6)	8.7 (<0.001)
Yes	65.4 (56.8–73.1)	58.7 (40.0–75.1)	6.7 (0.461)
Religion			
Catholics	51.3 (48.2–54.3)	45.3 (39.6–51.2)	6.0 (0.034)
Other Christians	45.3 (43.1–47.4)	45.2 (41.7–48.8)	0.1 (0.951)
Islam	39.7 (37.8–41.6)	26.2 (23.8–28.9)	13.5 (<0.001)
Others	41.1 (29.0–54.4)	26.8 (10.3–53.8)	14.3 (0.204)
Ethnicity			
Fulani	31.0 (26.5–35.8)	21.7 (15.3–29.8)	9.3 (<0.05)
Hausa	47.0 (44.3–49.7)	25.5 (22.6–28.6)	21.5 (<0.001)
Igbo	53.2 (50.5–55.9)	55.2 (49.4–60.9)	2.0 (<0.429)
Yoruba	44.7 (39.9–49.5)	38.9 (31.9–46.4)	5.8 (<0.05)
Other ethnic minorities	35.7 (33.7–37.8)	35.7 (32.4–39.0)	0.0 (1.000)
Marital status			
Never married	43.0 (41.4–44.6)	33.7 (31.7–35.7)	9.3 (<0.001)
Ever married	42.1 (40.0–44.3)	34.8 (28.1–42.1)	7.3 (<0.05)
Sex of household head			
Male	42.6 (41.1–44.0)	32.9 (30.8–35.2)	9.7 (<0.001)
Female	42.9 (40.1–45.7)	38.7 (34.2–43.5)	4.2 (0.069)
Currently working			
No	44.1 (42.5–45.8)	35.2 (32.0–38.6)	8.9 (<0.001)
Yes	40.7 (38.8–42.7)	32.9 (30.5–35.4)	7.8 (<0.001)
Ever had sex			
No	40.7 (38.9–42.5)	29.7 (27.6–31.8)	11.0 (<0.001)
Yes	44.0 (42.3–45.8)	46.8 (42.8–50.8)	−2.8 (0.098)

CI, confidence interval; *P_F_*, prevalence among women; *P_M_*, prevalence among men.

a *z* test of difference in two proportions.

The cRR and aRR obtained from the log-binomial regression models for the associated risk factors of comprehensive HIV knowledge are presented in Table 3. Comprehensive HIV knowledge was higher among women aged 18–19 (aRR = 1.09; 95% CI: 1.02–1.17) and 20–24 years (aRR = 1.20; 95% CI: 1.20–1.28) compared with women aged 15–17 years. Similarly, adolescent men aged 18–19 years (aRR = 1.22; 95% CI: 1.06–1.40) and male adults aged 20–24 years (aRR = 1.41; 95% CI: 1.23–1.63) had higher comprehensive HIV knowledge than men aged 15–17 years. The comprehensive knowledge about HIV was positively associated with wealth status; men in poorer, middle, richer and richest wealth quintiles were 1.34, 1.42, 1.56 and 2.04 times more likely to have comprehensive HIV knowledge than those from the poorest quintiles.

**Table 3.** Crude and adjusted log-binomial regression model of the factors associated with comprehensive knowledge of HIV among young women and men.

Variables	Female		Male	
	cRR (95% CI)	aRR (95% CI)	cRR (95% CI)	aRR (95% CI)
Current use of modern contraceptive				
No	Reference	Reference	Reference	Reference
Yes	1.32 (1.22–1.43)***	1.05 (0.98–1.14)	1.91 (1.70–2.14)***	1.23 (1.03–1.46)**
Age group				
15–17	Reference	Reference	Reference	Reference
18–19	1.20 (1.12–1.28)***	1.09 (1.02–1.17)**	1.37 (1.19–1.58)***	1.22 (1.06–1.40)**
20–24	1.36 (1.28–1.43)***	1.20 (1.12–1.28)**	1.65 (1.46–1.86)***	1.41 (1.23–1.63)***
Ethnicity				
North Central	Reference	Reference	Reference	Reference
North East	0.88 (0.77–1.01)*	1.19 (1.06–1.33)**	0.53 (0.43–0.66)***	0.67 (0.53–0.85)**
North West	1.58 (1.43–1.75)***	1.97 (1.77–2.20)***	0.68 (0.57–0.81)***	0.80 (0.63–1.03)*
South East	1.63 (1.47–1.82)***	1.12 (0.96–1.31)	1.36 (1.15–1.61)***	0.85 (0.66–1.09)
South South	1.22 (1.07–1.38)**	0.91 (0.81–1.02)*	1.12 (0.94–1.35)	0.78 (0.65–0.94)**
South West	1.40 (1.23–1.59)***	1.08 (0.95–1.23)	1.01 (0.82–1.26)	0.78 (0.60–1.00)*
Place of residence				
Urban	Reference	Reference	Reference	Reference
Rural	0.70 (0.65–0.74)***	0.87 (0.82–0.93)***	0.67 (0.60–0.75)***	0.96 (0.85–1.07)
Wealth quintiles				
Poorest	Reference	Reference	Reference	Reference
Poorer	1.16 (1.04–1.31)*	1.01 (0.92–1.12)	0.67 (0.60–0.75)***	1.34 (1.02–1.77)**
Middle	1.39 (1.23–1.58)***	1.10 (0.97–1.25)	1.78 (1.37–2.31)***	1.42 (1.03–1.96)**
Richer	1.59 (1.41–1.80)***	1.15 (1.01–1.29)**	2.67 (2.07–3.46)***	1.56 (1.14–2.13)**
Richest	1.87 (1.67–2.09)***	1.24 (1.09–1.42)**	3.62 (2.82–4.63)***	2.04 (1.47–2.83)***
Education				
None	Reference	Reference	Reference	Reference
Primary	1.09 (0.98–1.22)	1.15 (1.03–1.28)**	1.30 (0.95–1.79)	0.97 (0.70–1.35)
Secondary and above	1.60 (1.47–1.74)***	1.47 (1.34–1.61)***	2.50 (1.96–3.19)***	1.36 (1.02–1.82)**
Own a phone				
No	Reference	Reference	Reference	Reference
Yes	1.50 (1.43–1.58)***	1.15 (1.09–1.22)***	1.61 (1.43–1.81)***	1.04 (0.91–1.19)
Exposure to mass media				
No	Reference	Reference	Reference	Reference
Yes	1.50 (1.41–1.60)***	1.16 (1.09–1.23)***	1.91 (1.65–2.21)***	1.29 (1.10–1.51)**
Access to internet				
No	Reference	Reference	Reference	Reference
Yes	1.50 (1.42–1.60)***	1.11 (1.04–1.18)**	1.76 (1.58–1.96)***	1.08 (0.96–1.22)
Covered by health insurance				
No	Reference	Reference	Reference	Reference
Yes	1.55 (1.36–1.76)***	1.10 (0.96–1.24)	1.75 (1.27–2.41)**	0.93 (0.66–1.32)
Religion				
Catholics	Reference	Reference	Reference	Reference
Other Christians	0.88 (0.82–0.95)**	0.88 (0.81–0.95)**	0.99 (0.86–1.15)	1.10 (0.95–1.27)
Islam	0.77 (0.72–0.84)***	0.66 (0.58–0.74)***	0.58 (0.49–0.68)***	0.90 (0.71–1.13)
Others	0.80 (0.58–1.10)	0.99 (0.73–1.35)	0.59 (0.25–1.39)	1.00 (0.48–2.10)
Ethnicity				
Fulani	Reference	Reference	Reference	Reference
Hausa	1.52 (1.29–1.78)***	1.07 (0.92–1.23)	1.17 (0.82–1.68)	0.92 (0.65–1.30)
Igbo	1.72 (1.47–2.01)***	0.99 (0.80–1.21)	2.55 (1.80–3.62)***	1.10 (0.73–1.64)
Yoruba	1.44 (1.20–1.74)***	0.96 (0.78–1.18)	1.80 (1.22–2.64)**	0.83 (0.55–1.26)
Others	1.15 (0.98–1.35)*	0.95 (0.81–1.11)	1.65 (1.16–2.33)**	0.90 (0.64–1.26)
Marital status				
Never married	Reference	Reference	Reference	Reference
Ever married	0.98 (0.92–1.04)	1.04 (0.97–1.11)	1.03 (0.84–1.27)	1.01 (0.82–1.25)
Sex of household head				
Male	Reference	Reference	Reference	Reference
Female	1.01 (0.94–1.08)	0.94 (0.89–1.01)*	1.18 (1.03–1.35)**	0.95 (0.84–1.08)
Currently working				
No	Reference	Reference	Reference	Reference
Yes	0.92 (0.87–0.98)**	0.93 (0.88–0.97)**	0.93 (0.83–1.05)	1.09 (0.97–1.23)
Sexual initiation				
No	Reference	Reference	Reference	Reference
Yes	1.08 (1.02–1.14)	1.15 (1.08–1.23)***	1.58 (1.42–1.75)***	1.10 (0.96–1.25)

aRR, adjusted relative risk; CI, confidence interval; cRR, crude relative risk.

* *p* < 0.1; **p < 0.05; ***p < 0.001.

In the univariate analysis, comprehensive HIV knowledge was 32% and 91% higher among men and women who currently use a modern contraceptive, respectively. While in the adjusted model, comprehensive HIV knowledge was 23% significantly higher among men.

There was a higher likelihood of comprehensive HIV knowledge among young women (aRR = 1.16; 95% CI: 1.09–1.23) and men (aRR = 1.29; 95% CI: 1.10–1.51) who were exposed to mass media compared with those who were not. Compared with women who do not, women who had access to the internet (aRR = 1.11; 95% CI: 1.04–1.18), who owned a phone (aRR = 1.15; 95% CI: 1.09–1.22) and had initiated sex (aRR = 1.15; 95% CI: 1.08–1.23) were more likely to have comprehensive knowledge of HIV. Also, comprehensive knowledge of HIV was higher among young women residing in the North East (aRR = 1.19; 95% CI: 1.06–1.33) and North West (aRR = 1.97; 95% CI: 1.77–2.20) but lower among young men residing in the North East (aRR = 0.67; 95% CI: 0.53–0.85) and South–South (aRR = 0.78; 95% CI: 0.65–0.94) region of Nigeria. The likelihood of comprehensive HIV knowledge was significantly lower among female Muslims (aRR = 0.66; 95% CI: 0.38–0.74) and other Christians (aRR = 0.88; 95% CI: 0.81–0.95) compared with female Catholics. Similarly, women residing in rural areas were less likely to have comprehensive HIV knowledge than those in the urban area, whereas no statistically significant difference was observed among men.

## Discussion

This study investigated factors associated with comprehensive HIV knowledge among young people in Nigeria. This study found age to be positively associated with comprehensive HIV knowledge among young male and young female Nigerians; older participants were more likely to have high levels of comprehensive HIV knowledge. This finding confirms the results of similar studies, which observed that relatively older women had an increased likelihood of having comprehensive HIV knowledge compared with younger women in Ghana^16^ and Sudan.^17^ We also found that sexual initiation was associated with comprehensive HIV knowledge among women. That is, women who indicated that they ever had sex were more likely to have comprehensive HIV knowledge than those who did not. This not only was expected but also helps to explain the relationship between comprehensive HIV knowledge, age and sexual debut. Older women are more likely to have had sex, gotten pregnant and are more likely to have attended antenatal care in a Nigerian healthcare facility where they might have had HIV education from healthcare workers.^18^

In addition, as part of standard practices in most healthcare systems in Africa, HIV status is established during pregnancy.^19^ So it is not uncommon for older women who are more likely to have had a pregnancy or witnessed other members of the family go through pregnancy to be more knowledgeable either from their interactions with the healthcare system or as part of their experiential knowledge from social interactions.^16^ We noted that comprehensive knowledge of HIV was generally higher among women than among men. This is consistent with some evidence from Ghana.^20^ This may be linked to subjective individual health beliefs and sex-based health-seeking behaviour, which may portray that women are much more concerned about their health than men.^21^ As a result, men may require much more intense behavioural communication strategies that can propel them to be more concerned about the need to seek and retain more knowledge about important health issues, including HIV.

In addition, it was found that young women in the richer and richest quintiles of wealth and young men in the poorer, middle, richer and richest quintiles had higher odds of having comprehensive HIV knowledge than their counterparts in the poorest quintiles. These findings are consistent with the results of previous studies in SSA.^22–24^ A possible explanation of this relationship between wealth and HIV knowledge is that being wealthy is associated with access to different media (i.e. radio, television and newspaper) and modern technology, such as smartphones, as well as higher educational attainment, all of which increase the possibility of coming into contact with information about HIV/AIDS.^25,26^

In line with the observations made from the background characteristics and the relationships with HIV/AIDS in this study, it was evident that exposure to mass media, access to the internet and ownership of mobile phones increase the likelihood of comprehensive HIV knowledge among young women in Nigeria. The implication of this finding for health promotion in Nigeria is that public health programmes should focus on providing information on HIV/AIDS using different media because media exposure is known to be a common and cost-effective means of enhancing knowledge and changing health behaviours in a developing country setting.^27,28^

Consistent with previous studies,^16,23^ this study revealed that geographical location/setting is a significant predictor of comprehensive HIV knowledge among young people. There were regional variations in comprehensive HIV knowledge, with higher levels of knowledge observed among young women in the North East and North West regions. However, lower levels of comprehensive HIV knowledge were observed in the North East and South–South regions among young men. This regional variation is supported by previous research from Ghana^22^ and Ethiopia.^26^ The regional variation in comprehensive HIV knowledge among young Nigerians could be underscored by the variations in HIV/AIDS prevalence in Nigeria because regions with a high prevalence of HIV/AIDS are more likely to have been the targets of health promotion efforts. This is very important as the northern region suffers disparities in healthcare access^29,30^ and is hesitant to some health policies because of religious, cultural practices and interpretations, and level of education.^31–33^

Similarly, rural–urban variations in comprehensive HIV knowledge were observed in this study. Previous studies^16,34^ have shown that young women in urban areas have higher odds of having comprehensive HIV knowledge compared with those in rural areas. Young women in urban settlements have an increased likelihood of higher education, access to different media platforms, exposure to health information and a net positive effect of social networks.^24^ These factors could explain the increased odds of comprehensive HIV knowledge among urban female residents.^24^ The rural–urban disparity, like the regional variation observed in this study, calls for more targeting of rural areas and regions in Nigeria where comprehensive HIV knowledge is low to ensure the equitable distribution of comprehensive HIV knowledge.

Another finding from this study was that being a young female Catholic in Nigeria was associated with higher odds of comprehensive HIV knowledge than being a Muslim or belonging to another Christian faith. A possible explanation for this variation among young women of different religious backgrounds may be due to the variation in the adoption of mandatory premarital HIV testing in many African countries.^16,35^ Generally, churches were more inclined to adopt voluntary premarital testing.^35^

### Strengths and limitations of the study

The study has some identifiable strengths, including its rigorous analytical procedure and the use of a nationally representative sample to investigate such a sensitive issue among young people in Nigeria. These qualities make the findings and recommendations generalisable to all young people in Nigeria. Meanwhile, the study has some limitations. For instance, considering the sensitive nature of HIV and its associated misconceptions, it is possible that social desirability bias might have affected responses from some of the young people. Also, a cross-sectional study design was employed; hence, a causal effect cannot be established between the independent variables and comprehensive HIV knowledge. Besides, the number of women far exceeded the number of male participants who were included in the study. Finally, the study was limited to persons aged 15–24 years, and it is possible that the results would have varied if other age categories had been included.

## Conclusion

The study has policy and programme implications for public health interests as key findings revealed that the background characteristics of young people (age, education, religion, location and economic status) are of critical benefit for gaining comprehensive HIV knowledge in Nigeria. The observation that sexual initiation was associated with comprehensive HIV knowledge among women calls for comprehensive sex education among young people to ensure that they obtain correct information and knowledge from credible sources and not through experimentation with sex. The role of the mass media in preventing HIV cannot be overemphasised as it has been consistently proven that exposure to mass media, access to the internet and ownership of mobile phones are all associated with an increased likelihood of comprehensive HIV knowledge among young women in Nigeria. The key findings from this study imply that public health programmes in Nigeria should focus on providing information on HIV/AIDS using different approaches as well as health promotion and education strategies in the formal and informal sectors.

Because media exposure is a common and cost-effective way of public health promotion and education in modern times, emphasis could also be placed on using this channel to reach the target populations. Support from religious leaders through HIV prevention will be complimentary during premarital HIV counselling and testing. Thus, the Ministry of Health may have to collaborate with religious bodies, offer detailed training to religious leaders who provide premarital counselling and ensure that such knowledge is passed on during counselling. Further studies are required to highlight variation in hotspots, the impact of comprehensive HIV knowledge on HIV prevalence and other associated factors like power dynamics and vulnerability. Similarly, it is important to investigate why the incidence of HIV is higher among women with a higher prevalence of comprehensive HIV knowledge compared with their male counterparts.

## Declarations

**Ethics approval and consent to participate:** This study was based on a publicly available secondary cross-sectional survey dataset from the DHS Program (https://www.dhsprogram.com/). Therefore, no further ethical requirement was required for the use of the dataset.

**Consent for publication:** Not applicable.

**Availability of data and materials:** The dataset used for this study can be downloaded after due permission from the Demographic and Health Surveys (DHS) Program archive and can be accessed from https://www.dhsprogram.com/data/dataset/Nigeria_Standard-DHS_2018.cfm?flag=1.
